# A Digital Respiratory Ward in Leicester, Leicestershire, and Rutland, England, for Patients With COVID-19: Economic Evaluation of the Impact on Acute Capacity and Wider National Health Service Resource Use

**DOI:** 10.2196/47441

**Published:** 2024-02-13

**Authors:** Jim Swift, Noel O'Kelly, Chris Barker, Alex Woodward, Sudip Ghosh

**Affiliations:** 1 Spirit Health Leicester United Kingdom; 2 Leicestershire Partnership NHS Trust Leicester United Kingdom; 3 Department of Allied Health Sciences De Montford University Leicester United Kingdom

**Keywords:** Covid-19, telemedicine, digital technology, home transition, length of stay, cost-effectiveness analysis, cost, costs, economic, economics, telehealth, hospitalization, hospital, hospitals, hospitalizations, resource, resources, hospital stay, ward, wards, virtual care, remote care, financial, finance, finances, remote, respiratory, SARS-CoV-2, pulmonary, lung, lungs, service, services, delivery

## Abstract

**Background:**

The COVID-19 pandemic stressed global health care systems’ acute capacity and caused a diversion of resources from elective care to the treatment of acute respiratory disease. In preparing for a second wave of COVID-19 infections, England’s National Health Service (NHS) in Leicester, Leicestershire, and Rutland sought to protect acute capacity in the winter of 2020-2021. Their plans included the introduction of a digital ward where patients were discharged home early and supported remotely by community-based respiratory specialists, who were informed about patient health status by a digital patient monitoring system.

**Objective:**

The objective of the digital ward was to maintain acute capacity through safe, early discharge of patients with COVID-19 respiratory disease. The study objective was to establish what impact this digital ward had on overall NHS resource use.

**Methods:**

There were no expected differences in patient outcomes. A cost minimization was performed to demonstrate the impact on the NHS resource use from discharging patients into a digital COVID-19 respiratory ward, compared to acute care length of stay (LOS). This evaluation included all 310 patients enrolled in the service from November 2020 (service commencement) to November 2021. Two primary methods, along with sensitivity analyses, were used to help overcome the uncertainty associated with the estimated comparators for the observational data on COVID-19 respiratory acute LOS, compared with the actual LOS of the 279 (90%) patients who were not discharged on oxygen nor were in critical care. Historic comparative LOS and an ordinary least squares model based on local monthly COVID-19 respiratory median LOS were used as comparators. Actual comparator data were sourced for the 31 (10%) patients who were discharged home and into the digital ward for oxygen weaning. Resource use associated with delivering care in the digital ward was sourced from the digital system and respiratory specialists.

**Results:**

In the base case, the digital ward delivered estimated health care system savings of 846.5 bed-days and US $504,197 in net financial savings across the 2 key groups of patients—those on oxygen and those not on oxygen at acute discharge (both *P*<.001). The mean gross and net savings per patient were US $1850 and US $1626 in the base case, respectively, without including any savings associated with a potential reduction in readmissions. The 30-day readmission rate was 2.9%, which was below comparative data. The mean cost of the intervention was US $223.53 per patient, 12.1% of the estimated gross savings. It was not until the costs were increased and the effect reduced simultaneously by 78.4% in the sensitivity analysis that the intervention was no longer cost saving.

**Conclusions:**

The digital ward delivered increased capacity and substantial financial savings and did so with a high degree of confidence, at a very low absolute and relative cost.

## Introduction

### Overview

The COVID-19 pandemic stressed global health care systems, diverting resources from elective care and prioritizing the care of people with acute respiratory disease [1].

The introduction of a digital ward that commenced enrolling patients in November 2020 in Leicester, Leicestershire, and Rutland (LLR) was a key part of National Health Service (NHS) preparations for an expected surge in COVID-19 infections in the winter of 2020-2021.

The background to this decision was the impact of the first wave on LLR acute bed availability and the use of digital technology to remotely monitor patients to support their chronic conditions, both at the start of the COVID-19 pandemic and previously [2,3]. There was evidence to support the care of people after discharge with a high risk of readmission [2-4], and digitally supported patients with infectious respiratory disease had a reduced length of stay (LOS) [5-7].

The primary objectives for the COVID-19 digital ward were to maintain acute bed capacity through safe, early acute discharge and step-down into a specialist respiratory–managed service in the patients’ homes. Patients with COVID-19 respiratory infections were discharged into the care of the Leicester Partnership Trust (LPT) specialist respiratory team who were supported by digital technology (Clinitouch, Spirit Health), which electronically conveyed clinical observations and health status information to clinicians in an algorithm-based, traffic light (red, amber, and green)–prioritized basis. The standard operating procedures (service description), inclusion and exclusion criteria, objectives, and outcome measures for the digital ward are displayed on the internet [8], and more detail related to the intervention can be found in a study that regarded the first 65 patients that accessed the digital ward [9].

Paper-based, phone-based, digitally-based, and wearable device–based digital wards were used during COVID-19 as vehicles for admission avoidance and for stepping down patients who had been acutely admitted. There were mixed results [10].

The findings could have implications for future pandemics and how health systems allocate resources to better recover from the pandemic.

### Objective

The objective of this study was to demonstrate the impact of a digital COVID-19 ward on NHS resource use. Specifically, the study aimed to establish if the digital ward achieved its primary goal of freeing up beds and the extent to which it reduced or increased overall NHS resource use.

## Methods

### Participants

There were 310 patients admitted to the University Hospitals Leicester (UHL) NHS Trust with COVID-19 respiratory disease; they were either discharged home between November 2020 and November 2021 into a digital ward to support their oxygen weaning (31/310, 10%) or had not required oxygen at discharge and needed additional support to recover (279/310, 90%). The patients’ mean age was 55.0 (SD 13.7; median 56; range 22-86) years, 3.2% (n=10) of patients were 80 years or older, and 40.6% (n=143) of patients were female. No ethnicity, comorbidity, or socioeconomic status information was collected. Patients were given the option to go home early and be supported digitally when they met the inclusion criteria [8].

### Resource Use, Perspective, and Time Horizon

This study was based on observational data and represents a cost minimization of a service evaluation. All patients discharged into the digital ward had confirmed COVID-19–related respiratory disease. The Consolidated Health Economic Evaluation Reporting Standards (CHEERS) 2022 guidelines for economic evaluations were followed [11] (see Multimedia Appendix 1). This cost minimization analysis only evaluated NHS resource use and the outcomes of interest were acute bed-days saved, their costs offset, and the costs of the digital ward. It was considered that the digital ward would not deliver any change in patients’ long-term, health-related quality of life or health outcomes.

The perspective taken was that of the English NHS. Any savings would have been in the acute sector and additional costs in the intermediate care sector. The resource use only considered the digital ward costs and the costs of its comparator, acute hospital care.

The time horizon was slightly more than over 12 months, and no discounting of costs was conducted. All costs were in 2020-2021 pounds sterling and converted to US dollars.

### Resource Use Data Sources

Comparator data for patients not on oxygen with acute LOS were sourced from patients discharged immediately prior to the introduction of the digital ward in November 2020 and a published NHS data set [12]. The difference between the duration of the LOS for patients discharged into the digital ward for oxygen weaning and those discharged routinely were acquired from UHL by LPT and NHS X (now part of the NHS Transformation Directorate). Mean clinical consultation durations and staff seniority were sourced from LPT (both correspondence: JS). Individual acute LOS, readmissions data, and the cost of a day in a respiratory ward were sourced from UHL (correspondence: SG). Staff unit costs were sourced from the Personal Social Services Research Unit (PSSRU) data set for 2020-2021 [13].

There were 2 principal costs associated with participants’ stay in the digital ward: the duration of patients’ digital ward LOS, which influenced the costs of the digital technology and was calculated on a per diem basis; and the number and duration of respiratory specialist–patient digital contacts. The duration of the digital ward stays and the number of contacts were sourced from the digital technology database. The method for estimating the mean duration of a clinical contact was described in the paragraph above.

Resource use data in both units and costs can be found in Multimedia Appendix 1.

### Comparison of Acute Ward LOS Versus Actual and Imputed Comparators

There were 2 different populations discharged into the digital ward; those on oxygen at discharge and those who were not.

Those on oxygen were subjected to an analysis conducted by NHS X and LPT. They were discharged from the hospital, on average, 9.9 days earlier than similar patients who had not accessed the digital ward.

The first 65 patients not on oxygen discharged into the digital ward left acute care in 3.3 days, 2.2 days (40% relative reduction in LOS) earlier than controls who did not have the potential to access the digital ward [9]. The information on controls’ LOS (5.5 days) was sourced immediately prior to the digital ward’s introduction. This is an analysis of all patients admitted into the LLR COVID-19 digital ward prior to the end of November 2021 and prior to the availability of disease-modifying medicines that reduced acute COVID-19 illness severity. This study includes the first 65 patients.

### Data Used to Estimate LOS, Patients Not on Oxygen—Comparators

**Figure 1:**
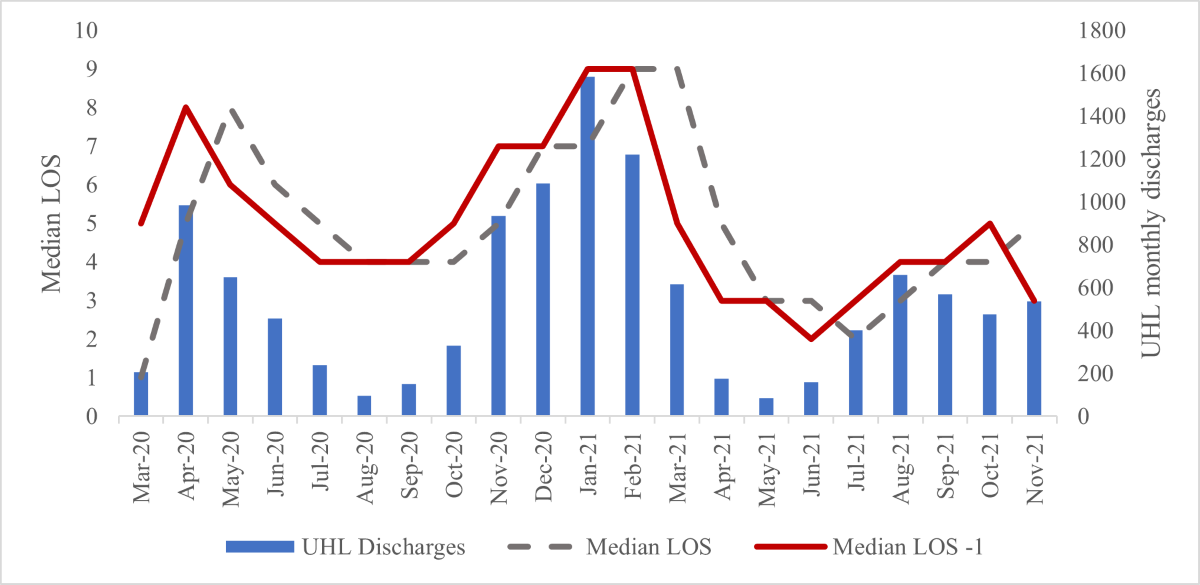
The relationship between median acute LOS and number of acute discharges in COVID-19 patients in UHL, March 2020-November 2021 ordinary least square model. LOS: length of stay; UHL: University Hospitals Leicester.

The LOS in UHL was reported alongside the median and mean monthly LOS for COVID-19 discharges between March 20 and December 21 [12]. The linear relationship between UHL discharges and median acute LOS is illustrated in Figure 1. The red line displays the result of pulling the data on the median LOS (gray hashed line) 1 month back in time. In doing so, the *r*^2^ improved from 0.31 to 0.75 in an ordinary least squares (OLS) model.

**Figure 2:**
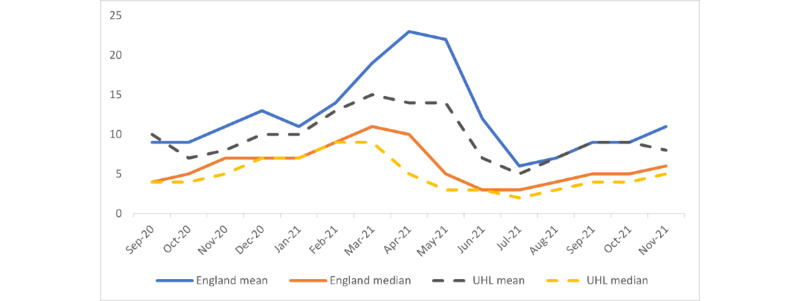
UHL and England monthly mean and median length of stay for patients with COVID-19: September 2020 to November 2021. UHL: University Hospitals Leicester.

Mean LOS included a minority of patients with intensive resource use and very long LOS, skewing the LOS upward (see Figure 2 for differences between mean and median LOS for England and UHL). The estimated comparator LOS was calculated for patients who were discharged without requiring oxygen. The authors considered that the median LOS better reflected patients’ estimated LOS.

Figure 2 shows the mean and median LOS for England and for UHL [12]. The solid blue and orange lines show the mean and median English LOS. The hashed gray and yellow lines show the mean and median UHL LOS.

### Patients Discharged Acutely Into the Digital Ward Not on Oxygen—Comparators

The 4 comparators below were then used to populate 2 simple simulations, creating 1000 random iterations for each of the 279 patients in both uniform and normal distributions, and the mean of both was taken as the base case.

1. An OLS regression was used to establish the quality of the relationship between monthly median LOS with the number of monthly acute discharges as the independent variable (*r*^2^=0.31; see Figure 1).
2. A regression similar to method 1 was used, but it used the better-fitted median data (1 month in arrears, *r*^2^=0.75). This improved relationship reflected that the data observed acute discharges instead of admissions in calendar months (see Figure 1).
3. The LOS in the comparator group in November 2020 was 5.5 days. This comparator assumed that LOS in November 2020 remained constant.
4. The comparator LOS data (5.5 days, prior to the introduction of the service) was 10% (5.0 days) longer than the median LOS for November 2020. This relative margin was continued in this median monthly LOS-based OLS comparator.

The data model used the 4 comparators and was conducted in Microsoft Excel. The *RANDBETWEEN* function was used to create a uniform model with 1000 iterations between the lowest and highest values of the 4 comparators for each patient’s LOS and the mean of the 1000 iterations was taken for all 279 patients. The same process was conducted to create a normal distribution of those data using the *NORMINV* and *RAND* functions, using the mean of the 4 comparators and their SD to develop 1000 iterations, from which the mean was taken. The final mean was taken from the 2 mean parameters and provided the base case.

### Patients Treated With Oxygen—Comparator

The NHS X and LPT analysis was used as the basis of the comparator for all patients treated with oxygen discharged into the digital ward.

### Sensitivity Analysis

A deterministic 1-way analysis was conducted to establish the extent to which varying the parameters influenced the savings. A 2-way analysis was developed to establish the extent to which the acute LOS and savings would have to be varied to reach the net savings threshold value of 0.

### Statistical Methods

The data sets were not normally distributed for both study subgroups (patients on and not on oxygen) on acute discharge. To evaluate the data, 2-tailed Wilcoxon signed-ranks tests for paired samples were used. Individual patients’ LOS was compared with their comparators. Normal approximation and ties correction were used, and α was set to .05. These methods were used consistently to compare paired data.

### Study Size

A total of 55 pairs were required for a 90% power to establish a difference between the 5.5-day LOS (the lowest comparator) for patients not on oxygen. The scale of the difference between the patients who were oxygen weaning meant that the number of pairs required was only 4. There were 310 patients in the study: 279 (90%) patients who were not discharged on oxygen and 31 (10%) patients who were discharged home and into the digital ward for oxygen weaning.

### Resource Use in the Digital Ward

Patients in the digital ward were monitored against a range of clinical criteria and classified by an algorithm into red, amber, and green ratings, which were highlighted on the LPT specialist respiratory clinicians’ dashboards. Red alerts provoked clinical contact within 24 hours throughout the patients’ stay in the digital ward; amber alerted the same, but in the first week only; and green rated patients who had not been contacted previously but were contacted in their second week in the digital ward. Patients were monitored using digital technology, which was billed on a per diem basis.

### Ethical Considerations

The study was evaluated by the Institutional Ethics Review body of DeMontfort University, with a decision reference of HLS FREC Ref: 2091/22. Approval was waived for the protocol as it was an economic analysis of a service that eligible patients routinely accessed as part of standard care. All analyzed data were deidentified. All patients accessed usual care. No payments were made to authors or patients. Patients or the public were not involved in the design, conduct, reporting, or dissemination plans of our research.

### Software Used for Analyses

All analyses were conducted in Microsoft Office Excel, Excel Analysis Toolpak, and The R Foundation’s R Studio.

## Results

### Overview

The 2 different patient populations had differing acute LOS. For patients not on oxygen when discharged, the mean acute LOS was 4.2 (SD 2.1; median 4.2) days. For patients treated with oxygen, the mean acute LOS was 13.3 (SD 4.5; median 12.4) days. Overall, the mean acute LOS was 4.3 (SD 2.1; median 4.4) days.

**Figure 3:**
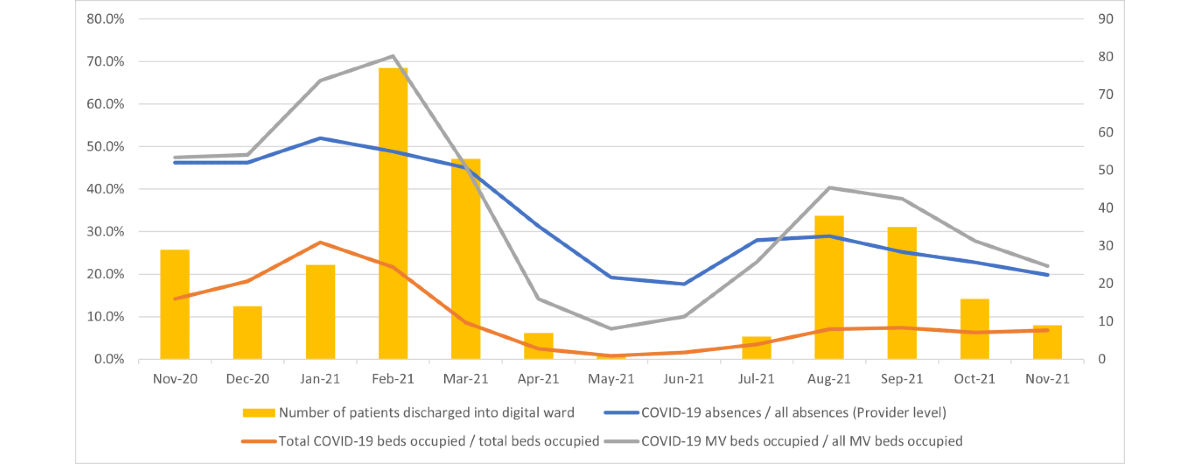
Digital ward admissions and supply stressors in University Hospitals Leicester between November 2020 and November 2021: acute beds, mechanically ventilated (MV) beds, and staff absences.

The phasing of patients discharged into the digital ward reflected demand in UHL [14]. The number of discharges into the COVID-19 ward (bars in Figure 3) corresponded with key system stressors (lines in Figure 3) rising and falling. The 3 key stressors reported were the ratios of COVID-19–related absences divided by all absences, total COVID-19–related beds occupied divided by all beds occupied, and COVID-19–related mechanically ventilated beds divided by all mechanically ventilated beds occupied [14].

### Patients Discharged Not on Oxygen—Acute LOS

Table 1 demonstrates the difference in acute LOS versus the imputed non–oxygen weaning LOS comparators.

The mean of the normal and uniform distributions from 1000 random iterations of the above data in each of the 279 non–oxygen weaning comparators were 6.31 and 5.95, respectively. The mean of both, 6.13, was used as the base case.

**Table 1:** Comparison of estimated comparators with acute University Hospitals Leicester length of stay prior to discharge into the COVID-19 digital ward in patients not weaning on oxygen between November 2020 and November 2021.

Wilcoxon signed rank tests with normal approximations and ties correction	Acute LOS^a^ prior to discharge to digital ward	Estimated LOS of comparator			
		Median LOS	5.5 days LOS	Modified median LOS	Median +10% LOS
W_min_^b^	N/A^c^	7192	6552.5	7355.5	5424.5
W (95% region of acceptance)	N/A	16,886.34-22,173.66	16,886.82-22,173.18	16,387.86-21,562.14	16,886.31-22,173.69
Z-statistic (zc=1.96)	N/A	–9.146816	–9.622666	–8.880232	–10.457076
Test *P* value	N/A	<.001	<.001	<.001	<.001
Mean	4.2	6.26	5.5	6.08	6.88

^a^LOS: length of stay.

^b^W_min_: Wilcoxon signed rank test statistic.

^c^N/A: not applicable.

A UK study of patients with COVID-19 not admitted to an intensive care unit estimated the acute LOS as 8.0 to 9.1 days [15]. All acute LOS comparators in the non–oxygen weaning group fell below this range, with the base case being almost 2 days beneath the lower value [15], supporting the conservatism of the model. The mean digital ward LOS was 14.2 days (SD 4.9; median 15) for patients not on oxygen.

### Patients Discharged on Oxygen—Acute LOS

The mean acute LOS for patients discharged into the digital ward on oxygen was 13.3 days, and the difference was 9.9 days (42.7%) between the actual LOS and the comparator LOS (*P*<.001). The mean digital ward LOS for patients on oxygen was 24.5 (SD 12.6; median 23) days.

### Digital Ward Resource Use

Digital ward resource use was driven by 2 elements: the number of clinical contacts and the LOS; the details are shown in Table 2. Multimedia Appendix 2 contains a breakdown of unit costs. How they were calculated is outlined in the *Methods* section.

**Table 2:** Total and per-patient costs of specialist respiratory consultations and digital technology in the COVID-19 digital ward between November 2020 and November 2021.

Specialist respiratory nurse or physiotherapist calls (n=310)	Contacts or days	Cost (US $)
Reds (total duration)	852	32,326
Ambers (week 1 only)	442	16,770
Greens (not previously contacted in week 1)	58	2201
Total consultations (at US $37.94 per contact)	1352	51,294
Number of digital ward days^a^	4711	17,999
Cost of clinical consultations per patient	N/A^b^	165.47
Monitoring costs per patient	N/A	58.06
Total patient contact and monitoring costs per patient	N/A	223.53

^a^Costs of Clinitouch per diem=US $3.82.

^b^N/A: not applicable.

### Estimated Resource Use and Savings

The range of estimated bed-days saved in the 279 patients not on oxygen varied from 363.8 (constant 5.5 days) to 749.9 (median OLS+10%); the comparison of digital ward patients versus all imputed controls was significant (*P*<.001). The base case was estimated to have saved 490.3 bed-days in the patients not on oxygen (*P*<.001). The estimated bed-days saved in the 31 patients on oxygen was 306.9, with which there was greater certainty (*P*<.001). The total estimated bed-days saved were between 670.7 and 1056.8 (2.2-3.4 days per patient). The base case estimate was 846 bed-days saved.

The cost of a bed day in a UHL acute respiratory ward in November 2020 was US $678.

Estimated implications on costs are summarized below:

- The total estimated gross savings related to bed-days were between US $454,404 and US $715,991 (US $1466-US $2310 per patient). The base case was US $573,490 (US $1850 per patient).
- Health care professional interventions were made to address patient symptoms. There were 1355 digital specialist respiratory consultations costing US $51,294 (US $165.47 per patient).
- The digital technology costs of the digital ward were US $17,999 (US $58.06 per patient).
- The total costs of the digital ward were US $69,293 (US $223.53 per patient).
- The total net savings were between US $385,411 and US $646,697 (US $1242-US $2086 per patient). The net savings in the base case were US $504,197 (US $1626 per patient).
- The intervention was cost saving in all scenarios. The costs of US $54,420 were between 9.7% (US $715,991) and 15.2% (US $454,404) of the estimated gross savings.

### Readmissions

There were 9 hospital readmissions (9/310, 2.9% of digital ward admissions) within 30 days. All readmissions were in the non–oxygen weaning cohort. In a systematic review, 10.3% (n=265,590) of those admitted with COVID-19 respiratory infections had a 30-day readmission [16]. Of the included English studies, 3 reported on 30-day readmission rates but included patients who had accessed critical care or had a high percentage of older adult patients [17-19] and reported readmission rates of 10.2% to 17.1%.

A total of 7.1% (n=154) of patients with mild or moderate COVID-19 disease, similar to the digital ward patients not on oxygen, discharged from a Turkish tertiary center were readmitted within 30 days [20]. Another systematic review [21] found that readmissions ranged from 4.2% [22] to 19.9% [23].

Evidence suggested that there was no difference between the rates of 30-day readmissions between the first and second COVID-19 waves [24]. If it was assumed the readmission rate for a comparator was 7.1% [20], the costs of readmissions would have been US $69,050 or 99.6% (US $69,293) of the total costs of running the digital ward. Using the lowest plausible comparator of 4.2% [22] would have offset 30.8% (US $21,342) of the costs of the digital ward. Any potential savings associated with readmissions were excluded from the results of the analysis because of the uncertainty associated with any potential comparator and to aid in the overall conservatism of this study.

### Estimated Carbon Dioxide Emissions

The carbon footprint associated with acutely hospitalized patients has been described as the most carbon-intensive care pathway and contributed 125 kg of carbon dioxide equivalent per day [25]. The gross reduction in 2019 carbon dioxide equivalent was 341 kg per patient in the base case and totaled 105.75 metric tonnes.

### Deterministic Sensitivity Analysis

There were 8 variables in this analysis; the differences in acute LOS for those who were on the digital ward versus comparators, the cost of an acute bed-day, the number of clinical contacts, the clinical contact duration, the cost per hour of clinicians, the digital ward LOS, and the digital technology costs.

A 1-way sensitivity analysis demonstrated that the 2 variables that changed by more than the relative input parameters were the LOS of patients who were and were not oxygen weaning. There was a linear 3.18% and 2.34% increase or decrease in the savings or costs for every 1% change in the value for the patients who did or did not access oxygen, respectively. All other resource-use variables increased or decreased at the same rate as the input variable. The parameter with the greatest uncertainty was the one that had the greatest impact on potential savings.

The cost of clinical consultations (respiratory specialists, LPT) and the cost of digital technology (Clinitouch, Spirit Health) were the 2 components of the digital ward costs. When the costs of both were simultaneously increased in a 2-way sensitivity analysis in the 279 patients not on oxygen by the same percentage as the LOS was reduced, it required a 75.4% change to reach the 0-threshold value. Performing the same for patients who accessed remote oxygen weaning, the threshold value was reached by simultaneously increasing costs and reducing the LOS cost offset by 87.5%. Overall, it took a reduction in effect and increase in costs of 78.4% (savings reduced from their estimated base case value of US $573,490 to 21.6% of that US $123,874 and costs to rise from US $69,293 to US $123,618) to reach a 0-threshold value.

## Discussion

Resource use in the digital ward was lower than all of the potential comparators. The net savings were estimated to have been between US $385,111 and US $646,697, with an estimated saving of US $1850 per patient in the base case. The digital ward costs were relatively low compared with the estimated gross savings (9.7% to 15.2%). The risk to health care systems of the digital ward not being cost saving was low. The UHL mean and median acute LOS were tracked at or below the England overall mean and median LOS between November 2020 and November 2021 (see Figure 2) [12]. The gap was largest after times of peak acute COVID-19 bed demand, which is also when the digital ward was used most to reduce pressure on UHL beds (see Figure 3). Both lend face validity to the findings. The COVID-19 digital ward intervention achieved its primary goal of increasing acute capacity. It also reduced overall NHS resource use, had a very low rate of readmissions, and reduced carbon dioxide emissions. Patients were released from acute wards earlier but clinically monitored for longer in the digital ward, which may have accounted for a plausible reduction in 30-day readmissions.

The main limitations associated with this analysis were the observational nature of the data and the associated use of imputed indirect comparators for 90% (279/310) of patients. The findings were most influenced by the parameter around which there was the greatest uncertainty. This renders a degree of uncertainty around the savings. Leicester City has a greater population of South Asian ethnicity than those of White ethnicity [26]. It is documented that people of South Asian ethnicity were at a higher risk of hospitalization from COVID-19 than their White counterparts [27]. The lack of socioeconomic status, comorbidities, and ethnicity data made the representativeness of the results difficult to interpret for wider settings.

The United Kingdom has a crisis in demand for elective care driven by an aging population [28]. It also had a shortfall in elective health care supply during the COVID-19 pandemic [1]. The supply-side constraint has been exacerbated by the lowest number of hospital beds per capita of all G7 countries [29] and social care’s inability to accommodate patients medically fit for discharge [30].

Specialist community respiratory teams exist widely across the United Kingdom. In addition, 82% and 99% of people older and younger than 55 years, respectively, owned a smartphone in the United Kingdom in 2022 [31]. The low financial and safety risk to health systems, digital scalability, widespread digital literacy, and high access to smartphones has meant there is an opportunity to be better prepared for future pandemics and enable wider digital diffusion into health care to support patients with other diseases, especially when there is continued capacity constraint. The findings were broadly consistent with other evaluations of digital wards where reductions in LOS were observed [32,33], and cost savings were found [32].

## Appendix

Multimedia Appendix 1 Consolidated Health Economic Evaluation Reporting Standards (CHEERS) 22 guidelines table.

Multimedia Appendix 2 Resource use unit tables.

Multimedia Appendix 3 Anonymized raw data file with original and comparator data.

## Abbreviations

CHEERS: consolidated health economic evaluation reporting standards
LLR: Leicester, Leicestershire, and Rutland
LOS: length of stay
LPT: Leicester Partnership Trust
NHS: National Health Service
OLS: ordinary least squares
PSSRU: Personal Social Services Research Unit
UHL: University Hospitals Leicester

## Data Availability

All data analyzed during this study are included in this published article and its supplementary information files. An anonymized data set is included in Multimedia Appendix 3.
