# Supplementary material for: A Digital Respiratory Ward in Leicester, Leicestershire, and Rutland, England, for Patients With COVID-19: Economic Evaluation of the Impact on Acute Capacity and Wider National Health Service Resource Use
Source: JMIR Form Res. 2024 Feb 13;8:e47441. doi: 10.2196/47441 (PMC10866202; doi:10.2196/47441)
Supplement: Multimedia Appendix 1 [file formative_v8i1e47441_app1.docx]

## Appendix 1 CHEERS 22 Guidelines

| 1 | Title | Title | Identify the study as an economic evaluation and specify the interventions being compared. | Pages: Title (1) and page 2 |
| --- | --- | --- | --- | --- |
| 2 | Abstract | Abstract | Provide a structured summary that highlights context, key methods, results, and alternative analyses. | Pages: Abstract (1) |
| 3 | Introduction | Background and objectives | Give the context for the study, the study question, and its practical relevance for decision making in policy or practice. | Pages: 2 |
| 4 | Methods | Health economic analysis plan | Indicate whether a health economic analysis plan was developed and where available. | N/A - CMA |
| 5 | Methods | Study population | Describe characteristics of the study population (such as age range, demographics, socioeconomic, or clinical characteristics). | Pages: 3 including limitations |
| 6 | Methods | Setting and location | Provide relevant contextual information that may influence findings. | Pages: 3 |
| 7 | Methods | Comparators | Describe the interventions or strategies being compared and why chosen. | Pages: 3, 4 |
| 8 | Methods | Perspective | State the perspective(s) adopted by the study and why chosen. | Page: 3 |
| 9 | Methods | Time horizon | State the time horizon for the study and why appropriate. | Page: 3 |
| 10 | Methods | Discount rate | Report the discount rate(s) and reason chosen. | Page: 3 |
| 11 | Methods | Selection of outcomes | Describe what outcomes were used as the measure(s) of benefit(s) and harm(s). | Page: 3 |
| 12 | Methods | Measurement of outcomes | Describe how outcomes used to capture benefit(s) and harm(s) were measured. | Page: 3 |
| 13 | Methods | Valuation of outcomes | Describe the population and methods used to measure and value outcomes. | Pages: 2, 3, 4 |
| 14 | Methods | Measurement and valuation of resources and costs | Describe how costs were valued. | Pages: 3 and appendix 1 |
| 15 | Methods | Currency, price date, and conversion | Report the dates of the estimated resource quantities and unit costs, plus the currency and year of conversion. | Page: 3 |
| 16 | Methods | Rationale and description of model | If modelling is used, describe in detail and why used. Report if the model is publicly available and where it can be accessed. | Pages: 3, 4 |
| 17 | Methods | Analytics and assumptions | Describe any methods for analysing or statistically transforming data, any extrapolation methods, and approaches for validating any model used. | Pages: 3, 4 |
| 18 | Methods | Characterising heterogeneity | Describe any methods used for estimating how the results of the study vary for subgroups. | Pages: 3, 4 |
| 19 | Methods | Characterising distributional effects | Describe how impacts are distributed across different individuals or adjustments made to reflect priority populations. | N/A |
| 20 | Methods | Characterising uncertainty | Describe methods to characterise any sources of uncertainty in the analysis. | Pages: 3, 4 |
| 21 | Methods | Approach to engagement with patients and others affected by the study | Describe any approaches to engage patients or service recipients, the general public, communities, or stakeholders (such as clinicians or payers) in the design of the study. | N/A |
| 22 | Results | Study parameters | Report all analytic inputs (such as values, ranges, references) including uncertainty or distributional assumptions. | Pages: 6,7 |
| 23 | Results | Summary of main results | Report the mean values for the main categories of costs and outcomes of interest and summarise them in the most appropriate overall measure. | Pages: 7,8,9 |
| 24 | Results | Effect of uncertainty | Describe how uncertainty about analytic judgments, inputs, or projections affect findings. Report the effect of choice of discount rate and time horizon, if applicable. | Pages: 9 |
| 25 | Results | Effect of engagement with patients and others affected by the study | Report on any difference patient/service recipient, general public, community, or stakeholder involvement made to the approach or findings of the study | N/A |
| 26 | Discussion | Study findings, limitations, generalisability, and current knowledge | Report key findings, limitations, ethical or equity considerations not captured, and how these could affect patients, policy, or practice. | Pages: 10 |
| 27 | Other relevant information | Source of funding | Describe how the study was funded and any role of the funder in the identification, design, conduct, and reporting of the analysis | Page 1 |
| 28 | Other relevant information | Conflicts of interest | Report authors conflicts of interest according to journal or International Committee of Medical Journal Editors requirements. | Page 1 |
