# Supplementary material for: A Digital Respiratory Ward in Leicester, Leicestershire, and Rutland, England, for Patients With COVID-19: Economic Evaluation of the Impact on Acute Capacity and Wider National Health Service Resource Use
Source: JMIR Form Res. 2024 Feb 13;8:e47441. doi: 10.2196/47441 (PMC10866202; doi:10.2196/47441)
Supplement: Multimedia Appendix 2 [file formative_v8i1e47441_app2.docx]

Multimedia Appendix 2 Resource Use Tables

Resource Use Unit Costs

*Table S1: Staff unit costs*

| Staff costs | Unit Cost £ |
| --- | --- |
| Band 7 nurse / physio per hour | £65 |

*Table S2: Staff time per consultation*

| Consultation duration | Time in minutes |
| --- | --- |
| Time for consultation 1 | 20 |
| Time for consultation 2 (follow up) | 7.5 |
| Total time | 27.5 |

*Table S3: Staff costs per consultation*

| Nurse / Physio call costs – RAG | Costs per virtual consultation |
| --- | --- |
| Band 7 | £29.79 |

*Table S4: Costs of digital technology per day*

| Costs of clinitouch | Clinitouch per diem |
| --- | --- |
| Rate | £3 |

*Table S5: Costs of acute ward stay UHL*

| Costs of respiratory ward day | Acute ward per diem |
| --- | --- |
| Rate | £532 |

Virtual Ward Resource Use and Costs

Resource Use Summaries in Units and Costs

*Table S6: Breakdown in Resource Use in the virtual ward in units*

| Resource Use | Number of Patients | Reduction in LOS (Days) | No. of days in VW | Red Alerts | Amber Alerts Week1 | Green Alerts Week2 |
| --- | --- | --- | --- | --- | --- | --- |
| O2 | 31 | 306.9 | 759 | 257 | 52 | 2 |
| Non-O2* | 279 | 539.6 | 3,952 | 595 | 390 | 56 |
| Combined | 310 | 846.5 | 4,711 | 852 | 442 | 58 |

*Table S7: Breakdown in Resource Use in The Virtual Ward in £s [Base Case]*

| Resource Use | Reduction in LOS (Days) | Costs of Digital | Costs of Virtual Consults | Total Costs | Net Savings |
| --- | --- | --- | --- | --- | --- |
| O2 | £163,271 | £2,277 | £8,481 | £10,758 | £152,513 |
| Non-O2* | £287,051 | £11,856 | £28,389 | £40,245 | £246,806 |
| Combined | £450,322 | £14,133 | £40,278 | £54,411 | £395,911 |

*Table S8: Breakdown in Resource Use in The Virtual Ward in £s per patient*

| Mean Resource Use per Patient | Reduction in LOS (Days) | Costs of Digital | Costs of Virtual Consults | Total Costs | Net Savings |
| --- | --- | --- | --- | --- | --- |
| O2 | £5,267 | £73 | £274 | £347 | £4,920 |
| Non-O2* | £1,029 | £42 | £102 | £144 | £885 |
| Combined | £1,453 | £46 | £130 | £176 | £1,277 |

*Using the base case for resource use in the non-O_2_ population.
