# Supplementary material for: A Digital Respiratory Ward in Leicester, Leicestershire, and Rutland, England, for Patients With COVID-19: Economic Evaluation of the Impact on Acute Capacity and Wider National Health Service Resource Use
Source: JMIR Form Res. 2024 Feb 13;8:e47441. doi: 10.2196/47441 (PMC10866202; doi:10.2196/47441)
Supplement: Multimedia Appendix 3 [file formative_v8i1e47441_app3.docx]

# Appendix 3 – Anonymised datasets

3a - Dataset Non-O2 accessing patients – RAG ratings & virtual ward LOS (ordered by acute LOS)

| Random ID | Number Of Red Sessions | Number Of Amber Sessions | Number Of Green Sessions | Total RAG Reports | Days In Virtual Ward |
| --- | --- | --- | --- | --- | --- |
| 1 | 0 | 0 | 22 | 22 | 17 |
| 2 | 2 | 5 | 9 | 16 | 16 |
| 3 | 0 | 1 | 13 | 14 | 15 |
| 4 | 0 | 0 | 15 | 15 | 15 |
| 5 | 0 | 1 | 13 | 14 | 15 |
| 6 | 0 | 1 | 11 | 12 | 5 |
| 7 | 11 | 2 | 3 | 16 | 16 |
| 8 | 1 | 8 | 6 | 15 | 15 |
| 9 | 0 | 0 | 15 | 15 | 16 |
| 10 | 0 | 0 | 15 | 15 | 15 |
| 11 | 0 | 0 | 19 | 19 | 16 |
| 12 | 0 | 0 | 16 | 16 | 20 |
| 13 | 1 | 0 | 11 | 12 | 14 |
| 14 | 2 | 6 | 5 | 13 | 13 |
| 15 | 5 | 5 | 8 | 18 | 16 |
| 16 | 2 | 3 | 12 | 17 | 17 |
| 17 | 14 | 0 | 1 | 15 | 16 |
| 18 | 2 | 1 | 11 | 14 | 13 |
| 19 | 1 | 1 | 13 | 15 | 15 |
| 20 | 1 | 1 | 8 | 10 | 9 |
| 21 | 4 | 1 | 9 | 14 | 14 |
| 22 | 0 | 0 | 15 | 15 | 15 |
| 23 | 0 | 8 | 7 | 15 | 18 |
| 24 | 5 | 10 | 1 | 16 | 15 |
| 25 | 3 | 6 | 6 | 15 | 17 |
| 26 | 0 | 2 | 13 | 15 | 15 |
| 27 | 0 | 0 | 1 | 1 | 1 |
| 28 | 0 | 0 | 1 | 1 | 1 |
| 29 | 0 | 0 | 15 | 15 | 16 |
| 30 | 1 | 6 | 9 | 16 | 15 |
| 31 | 2 | 18 | 3 | 23 | 23 |
| 32 | 0 | 3 | 11 | 14 | 15 |
| 33 | 9 | 0 | 0 | 9 | 12 |
| 34 | 15 | 1 | 0 | 16 | 16 |
| 35 | 9 | 6 | 2 | 17 | 19 |
| 36 | 2 | 11 | 2 | 15 | 15 |
| 37 | 0 | 1 | 16 | 17 | 17 |
| 38 | 0 | 0 | 16 | 16 | 16 |
| 39 | 1 | 2 | 10 | 13 | 16 |
| 40 | 3 | 2 | 11 | 16 | 16 |
| 41 | 0 | 1 | 14 | 15 | 17 |
| 42 | 1 | 2 | 11 | 14 | 14 |
| 43 | 1 | 4 | 10 | 15 | 15 |
| 44 | 1 | 6 | 9 | 16 | 16 |
| 45 | 15 | 0 | 0 | 15 | 15 |
| 46 | 11 | 1 | 0 | 12 | 16 |
| 47 | 1 | 0 | 14 | 15 | 15 |
| 48 | 0 | 0 | 16 | 16 | 16 |
| 49 | 4 | 2 | 9 | 15 | 15 |
| 50 | 1 | 3 | 11 | 15 | 16 |
| 51 | 1 | 2 | 12 | 15 | 18 |
| 52 | 0 | 1 | 5 | 6 | 6 |
| 53 | 0 | 3 | 12 | 15 | 15 |
| 54 | 1 | 2 | 13 | 16 | 17 |
| 55 | 3 | 9 | 3 | 15 | 15 |
| 56 | 0 | 1 | 17 | 18 | 16 |
| 57 | 1 | 0 | 13 | 14 | 16 |
| 58 | 0 | 1 | 0 | 1 | 1 |
| 59 | 4 | 7 | 2 | 13 | 14 |
| 60 | 4 | 3 | 9 | 16 | 16 |
| 61 | 7 | 7 | 0 | 14 | 15 |
| 62 | 0 | 1 | 0 | 1 | 1 |
| 63 | 8 | 6 | 1 | 15 | 15 |
| 64 | 0 | 0 | 16 | 16 | 16 |
| 65 | 1 | 4 | 10 | 15 | 15 |
| 66 | 0 | 0 | 1 | 1 | 1 |
| 67 | 0 | 1 | 16 | 17 | 17 |
| 68 | 6 | 8 | 2 | 16 | 8 |
| 69 | 0 | 1 | 13 | 14 | 15 |
| 70 | 1 | 3 | 13 | 17 | 16 |
| 71 | 18 | 12 | 1 | 31 | 33 |
| 72 | 8 | 6 | 1 | 15 | 16 |
| 73 | 0 | 3 | 7 | 10 | 18 |
| 74 | 0 | 4 | 9 | 13 | 15 |
| 75 | 0 | 1 | 13 | 14 | 15 |
| 76 | 0 | 1 | 15 | 16 | 16 |
| 77 | 0 | 1 | 11 | 12 | 16 |
| 78 | 1 | 5 | 9 | 15 | 18 |
| 79 | 2 | 0 | 13 | 15 | 16 |
| 80 | 0 | 3 | 9 | 12 | 17 |
| 81 | 3 | 7 | 6 | 16 | 16 |
| 82 | 2 | 0 | 0 | 2 | 2 |
| 83 | 2 | 4 | 10 | 16 | 15 |
| 84 | 9 | 2 | 2 | 13 | 19 |
| 85 | 0 | 3 | 12 | 15 | 16 |
| 86 | 1 | 2 | 11 | 14 | 14 |
| 87 | 0 | 3 | 10 | 13 | 15 |
| 88 | 0 | 0 | 16 | 16 | 16 |
| 89 | 0 | 0 | 16 | 16 | 16 |
| 90 | 2 | 5 | 13 | 20 | 14 |
| 91 | 0 | 1 | 15 | 16 | 16 |
| 92 | 13 | 0 | 1 | 14 | 14 |
| 93 | 0 | 0 | 1 | 1 | 1 |
| 94 | 0 | 2 | 12 | 14 | 14 |
| 95 | 4 | 3 | 13 | 20 | 21 |
| 96 | 0 | 1 | 12 | 13 | 14 |
| 97 | 0 | 0 | 16 | 16 | 19 |
| 98 | 3 | 1 | 13 | 17 | 16 |
| 99 | 0 | 0 | 7 | 7 | 7 |
| 100 | 6 | 9 | 1 | 16 | 16 |
| 101 | 1 | 7 | 8 | 16 | 16 |
| 102 | 1 | 0 | 16 | 17 | 18 |
| 103 | 15 | 1 | 1 | 17 | 14 |
| 104 | 0 | 0 | 14 | 14 | 15 |
| 105 | 0 | 2 | 1 | 3 | 5 |
| 106 | 0 | 1 | 8 | 9 | 9 |
| 107 | 1 | 0 | 15 | 16 | 16 |
| 108 | 0 | 0 | 15 | 15 | 15 |
| 109 | 0 | 0 | 14 | 14 | 14 |
| 110 | 0 | 5 | 8 | 13 | 14 |
| 111 | 0 | 14 | 1 | 15 | 15 |
| 112 | 0 | 5 | 12 | 17 | 14 |
| 113 | 3 | 0 | 10 | 13 | 13 |
| 114 | 0 | 1 | 11 | 12 | 13 |
| 115 | 0 | 0 | 1 | 1 | 1 |
| 116 | 0 | 14 | 0 | 14 | 15 |
| 117 | 13 | 3 | 1 | 17 | 29 |
| 118 | 1 | 5 | 10 | 16 | 17 |
| 119 | 0 | 0 | 15 | 15 | 15 |
| 120 | 0 | 0 | 15 | 15 | 15 |
| 121 | 0 | 4 | 11 | 15 | 16 |
| 122 | 1 | 0 | 15 | 16 | 16 |
| 123 | 27 | 3 | 0 | 30 | 30 |
| 124 | 0 | 0 | 5 | 5 | 8 |
| 125 | 1 | 0 | 15 | 16 | 16 |
| 126 | 0 | 0 | 1 | 1 | 1 |
| 127 | 1 | 2 | 12 | 15 | 16 |
| 128 | 1 | 2 | 13 | 16 | 16 |
| 129 | 5 | 2 | 1 | 8 | 7 |
| 130 | 0 | 0 | 15 | 15 | 15 |
| 131 | 1 | 0 | 10 | 11 | 15 |
| 132 | 10 | 10 | 2 | 22 | 22 |
| 133 | 4 | 8 | 4 | 16 | 18 |
| 134 | 2 | 6 | 7 | 15 | 14 |
| 135 | 0 | 4 | 5 | 9 | 16 |
| 136 | 0 | 0 | 1 | 1 | 1 |
| 137 | 0 | 0 | 3 | 3 | 6 |
| 138 | 0 | 0 | 14 | 14 | 16 |
| 139 | 1 | 1 | 12 | 14 | 15 |
| 140 | 0 | 0 | 15 | 15 | 16 |
| 141 | 10 | 4 | 1 | 15 | 15 |
| 142 | 0 | 0 | 16 | 16 | 15 |
| 143 | 13 | 2 | 0 | 15 | 15 |
| 144 | 0 | 1 | 0 | 1 | 1 |
| 145 | 7 | 0 | 7 | 14 | 14 |
| 146 | 0 | 0 | 17 | 17 | 16 |
| 147 | 0 | 0 | 16 | 16 | 17 |
| 148 | 4 | 3 | 9 | 16 | 16 |
| 149 | 2 | 2 | 4 | 8 | 9 |
| 150 | 1 | 2 | 12 | 15 | 16 |
| 151 | 2 | 2 | 10 | 14 | 16 |
| 152 | 0 | 0 | 14 | 14 | 14 |
| 153 | 0 | 0 | 2 | 2 | 2 |
| 154 | 0 | 1 | 12 | 13 | 14 |
| 155 | 0 | 1 | 17 | 18 | 16 |
| 156 | 1 | 0 | 0 | 1 | 1 |
| 157 | 1 | 0 | 5 | 6 | 6 |
| 158 | 0 | 0 | 13 | 13 | 14 |
| 159 | 1 | 3 | 11 | 15 | 14 |
| 160 | 0 | 2 | 15 | 17 | 16 |
| 161 | 0 | 4 | 11 | 15 | 15 |
| 162 | 3 | 4 | 10 | 17 | 16 |
| 163 | 1 | 2 | 10 | 13 | 14 |
| 164 | 1 | 0 | 14 | 15 | 15 |
| 165 | 1 | 1 | 14 | 16 | 16 |
| 166 | 1 | 0 | 14 | 15 | 15 |
| 167 | 0 | 1 | 0 | 1 | 1 |
| 168 | 0 | 0 | 15 | 15 | 18 |
| 169 | 2 | 6 | 3 | 11 | 16 |
| 170 | 4 | 0 | 12 | 16 | 16 |
| 171 | 0 | 13 | 10 | 23 | 21 |
| 172 | 2 | 1 | 10 | 13 | 14 |
| 173 | 0 | 10 | 4 | 14 | 20 |
| 174 | 0 | 3 | 12 | 15 | 16 |
| 175 | 1 | 1 | 13 | 15 | 27 |
| 176 | 0 | 0 | 16 | 16 | 16 |
| 177 | 0 | 0 | 14 | 14 | 15 |
| 178 | 0 | 7 | 5 | 12 | 13 |
| 179 | 3 | 1 | 7 | 11 | 16 |
| 180 | 0 | 0 | 14 | 14 | 14 |
| 181 | 0 | 2 | 3 | 5 | 6 |
| 182 | 0 | 5 | 12 | 17 | 17 |
| 183 | 0 | 0 | 13 | 13 | 15 |
| 184 | 1 | 2 | 8 | 11 | 15 |
| 185 | 1 | 1 | 13 | 15 | 14 |
| 186 | 0 | 0 | 1 | 1 | 17 |
| 187 | 8 | 5 | 1 | 14 | 15 |
| 188 | 0 | 2 | 13 | 15 | 15 |
| 189 | 1 | 3 | 13 | 17 | 15 |
| 190 | 4 | 0 | 11 | 15 | 15 |
| 191 | 14 | 2 | 0 | 16 | 16 |
| 192 | 1 | 1 | 13 | 15 | 15 |
| 193 | 4 | 4 | 5 | 13 | 14 |
| 194 | 3 | 1 | 9 | 13 | 15 |
| 195 | 0 | 2 | 11 | 13 | 14 |
| 196 | 0 | 0 | 15 | 15 | 16 |
| 197 | 0 | 0 | 5 | 5 | 17 |
| 198 | 0 | 5 | 7 | 12 | 15 |
| 199 | 0 | 1 | 15 | 16 | 15 |
| 200 | 14 | 0 | 0 | 14 | 14 |
| 201 | 0 | 5 | 12 | 17 | 16 |
| 202 | 0 | 0 | 1 | 1 | 1 |
| 203 | 3 | 8 | 1 | 12 | 14 |
| 204 | 0 | 1 | 0 | 1 | 1 |
| 205 | 0 | 5 | 11 | 16 | 16 |
| 206 | 1 | 3 | 1 | 5 | 4 |
| 207 | 3 | 4 | 8 | 15 | 16 |
| 208 | 1 | 6 | 9 | 16 | 17 |
| 209 | 1 | 2 | 11 | 14 | 14 |
| 210 | 0 | 2 | 13 | 15 | 15 |
| 211 | 1 | 3 | 11 | 15 | 15 |
| 212 | 21 | 0 | 0 | 21 | 21 |
| 213 | 5 | 1 | 8 | 14 | 15 |
| 214 | 1 | 0 | 0 | 1 | 1 |
| 215 | 1 | 4 | 10 | 15 | 14 |
| 216 | 5 | 5 | 3 | 13 | 13 |
| 217 | 0 | 3 | 6 | 9 | 10 |
| 218 | 3 | 2 | 4 | 9 | 10 |
| 219 | 2 | 2 | 13 | 17 | 17 |
| 220 | 1 | 2 | 5 | 8 | 8 |
| 221 | 1 | 0 | 4 | 5 | 7 |
| 222 | 10 | 4 | 1 | 15 | 18 |
| 223 | 1 | 3 | 11 | 15 | 15 |
| 224 | 0 | 4 | 10 | 14 | 15 |
| 225 | 0 | 2 | 13 | 15 | 15 |
| 226 | 0 | 4 | 12 | 16 | 19 |
| 227 | 3 | 4 | 8 | 15 | 15 |
| 228 | 0 | 2 | 14 | 16 | 17 |
| 229 | 1 | 0 | 15 | 16 | 17 |
| 230 | 0 | 2 | 12 | 14 | 12 |
| 231 | 0 | 4 | 8 | 12 | 13 |
| 232 | 0 | 0 | 12 | 12 | 12 |
| 233 | 0 | 0 | 16 | 16 | 18 |
| 234 | 7 | 13 | 1 | 21 | 21 |
| 235 | 2 | 1 | 14 | 17 | 18 |
| 236 | 15 | 0 | 0 | 15 | 15 |
| 237 | 0 | 0 | 16 | 16 | 16 |
| 238 | 0 | 0 | 15 | 15 | 15 |
| 239 | 10 | 3 | 0 | 13 | 14 |
| 240 | 0 | 0 | 15 | 15 | 15 |
| 241 | 2 | 6 | 8 | 16 | 15 |
| 242 | 13 | 0 | 9 | 22 | 22 |
| 243 | 2 | 3 | 10 | 15 | 17 |
| 244 | 1 | 0 | 14 | 15 | 15 |
| 245 | 0 | 0 | 5 | 5 | 8 |
| 246 | 1 | 3 | 10 | 14 | 15 |
| 247 | 4 | 3 | 9 | 16 | 15 |
| 248 | 0 | 1 | 10 | 11 | 13 |
| 249 | 3 | 9 | 2 | 14 | 15 |
| 250 | 4 | 0 | 1 | 5 | 8 |
| 251 | 1 | 3 | 0 | 4 | 6 |
| 252 | 0 | 0 | 13 | 13 | 13 |
| 253 | 0 | 1 | 14 | 15 | 15 |
| 254 | 0 | 0 | 17 | 17 | 16 |
| 255 | 0 | 0 | 16 | 16 | 17 |
| 256 | 2 | 4 | 6 | 12 | 16 |
| 257 | 0 | 8 | 8 | 16 | 16 |
| 258 | 0 | 2 | 7 | 9 | 15 |
| 259 | 0 | 2 | 3 | 5 | 8 |
| 260 | 1 | 1 | 12 | 14 | 15 |
| 261 | 1 | 2 | 13 | 16 | 16 |
| 262 | 0 | 0 | 15 | 15 | 15 |
| 263 | 1 | 0 | 0 | 1 | 1 |
| 264 | 0 | 3 | 11 | 14 | 14 |
| 265 | 1 | 4 | 9 | 14 | 15 |
| 266 | 0 | 3 | 10 | 13 | 15 |
| 267 | 0 | 2 | 14 | 16 | 15 |
| 268 | 0 | 3 | 15 | 18 | 16 |
| 269 | 0 | 7 | 9 | 16 | 14 |
| 270 | 6 | 0 | 10 | 16 | 16 |
| 271 | 2 | 0 | 19 | 21 | 21 |
| 272 | 1 | 0 | 0 | 1 | 1 |
| 273 | 0 | 0 | 11 | 11 | 12 |
| 274 | 2 | 2 | 12 | 16 | 16 |
| 275 | 0 | 1 | 20 | 21 | 18 |
| 276 | 4 | 8 | 2 | 14 | 14 |
| 277 | 1 | 0 | 1 | 2 | 3 |
| 278 | 0 | 2 | 12 | 14 | 16 |
| 279 | 0 | 1 | 13 | 14 | 15 |
|  | 595 | 671 | 2489 | 3755 | 3952 |

Appendix 3 – Anonymised datasets

3b - Dataset Non-O2 accessing patients – Acute LOS (ordered by acute LOS)

| Random ID | Length of Ward Stay prior to discharge to virtual ward | Estimated LOS of comparator (Median LOS) | Estimated LOS of comparator (5.5 LOS) | Estimated LOS of comparator (Modified median LOS) | Estimated LOS of comparator (Median +10% LOS) |
| --- | --- | --- | --- | --- | --- |
| 1 | 14 | 9 | 5.5 | 9 | 9.9 |
| 2 | 12.7 | 4 | 5.5 | 4 | 4.4 |
| 3 | 12 | 9 | 5.5 | 9 | 9.9 |
| 4 | 10.5 | 3 | 5.5 | 4 | 3.3 |
| 5 | 10.5 | 4 | 5.5 | 4 | 4.4 |
| 6 | 10.5 | 4 | 5.5 | 5 | 4.4 |
| 7 | 9.8 | 7 | 5.5 | 7 | 7.7 |
| 8 | 9.4 | 9 | 5.5 | 9 | 9.9 |
| 9 | 9.4 | 5 | 5.5 | 3 | 5.5 |
| 10 | 8.4 | 7 | 5.5 | 7 | 7.7 |
| 11 | 8.3 | 3 | 5.5 | 4 | 3.3 |
| 12 | 8.3 | 4 | 5.5 | 4 | 4.4 |
| 13 | 8.3 | 4 | 5.5 | 5 | 4.4 |
| 14 | 8.1 | 7 | 5.5 | 9 | 7.7 |
| 15 | 7.9 | 9 | 5.5 | 9 | 9.9 |
| 16 | 7.8 | 9 | 5.5 | 9 | 9.9 |
| 17 | 7.4 | 9 | 5.5 | 9 | 9.9 |
| 18 | 7.4 | 3 | 5.5 | 4 | 3.3 |
| 19 | 7.4 | 3 | 5.5 | 4 | 3.3 |
| 20 | 7.4 | 4 | 5.5 | 4 | 4.4 |
| 21 | 7.4 | 4 | 5.5 | 4 | 4.4 |
| 22 | 7.2 | 9 | 5.5 | 5 | 9.9 |
| 23 | 6.5 | 9 | 5.5 | 9 | 9.9 |
| 24 | 6.5 | 9 | 5.5 | 9 | 9.9 |
| 25 | 6.5 | 9 | 5.5 | 5 | 9.9 |
| 26 | 6.5 | 2 | 5.5 | 3 | 2.2 |
| 27 | 6.5 | 3 | 5.5 | 4 | 3.3 |
| 28 | 6.5 | 4 | 5.5 | 4 | 4.4 |
| 29 | 6.4 | 9 | 5.5 | 9 | 9.9 |
| 30 | 6.4 | 9 | 5.5 | 9 | 9.9 |
| 31 | 6.4 | 9 | 5.5 | 9 | 9.9 |
| 32 | 6.4 | 9 | 5.5 | 5 | 9.9 |
| 33 | 6.4 | 9 | 5.5 | 5 | 9.9 |
| 34 | 6.4 | 3 | 5.5 | 4 | 3.3 |
| 35 | 6.4 | 3 | 5.5 | 4 | 3.3 |
| 36 | 6.4 | 4 | 5.5 | 4 | 4.4 |
| 37 | 6.4 | 4 | 5.5 | 4 | 4.4 |
| 38 | 6.4 | 4 | 5.5 | 4 | 4.4 |
| 39 | 6.2 | 5 | 5.5 | 7 | 5.5 |
| 40 | 6.2 | 9 | 5.5 | 9 | 9.9 |
| 41 | 6.2 | 9 | 5.5 | 9 | 9.9 |
| 42 | 6.1 | 5 | 5.5 | 3 | 5.5 |
| 43 | 6.1 | 4 | 5.5 | 5 | 4.4 |
| 44 | 6 | 9 | 5.5 | 9 | 9.9 |
| 45 | 5.9 | 9 | 5.5 | 9 | 9.9 |
| 46 | 5.9 | 9 | 5.5 | 5 | 9.9 |
| 47 | 5.9 | 5 | 5.5 | 3 | 5.5 |
| 48 | 5.8 | 9 | 5.5 | 9 | 9.9 |
| 49 | 5.8 | 9 | 5.5 | 5 | 9.9 |
| 50 | 5.8 | 5 | 5.5 | 3 | 5.5 |
| 51 | 5.7 | 9 | 5.5 | 9 | 9.9 |
| 52 | 5.7 | 4 | 5.5 | 4 | 4.4 |
| 53 | 5.4 | 9 | 5.5 | 9 | 9.9 |
| 54 | 5.4 | 9 | 5.5 | 9 | 9.9 |
| 55 | 5.4 | 9 | 5.5 | 9 | 9.9 |
| 56 | 5.4 | 9 | 5.5 | 5 | 9.9 |
| 57 | 5.4 | 9 | 5.5 | 5 | 9.9 |
| 58 | 5.4 | 9 | 5.5 | 5 | 9.9 |
| 59 | 5.4 | 5 | 5.5 | 3 | 5.5 |
| 60 | 5.4 | 3 | 5.5 | 4 | 3.3 |
| 61 | 5.4 | 3 | 5.5 | 4 | 3.3 |
| 62 | 5.4 | 4 | 5.5 | 4 | 4.4 |
| 63 | 5.4 | 4 | 5.5 | 4 | 4.4 |
| 64 | 5.2 | 3 | 5.5 | 4 | 3.3 |
| 65 | 5.2 | 4 | 5.5 | 4 | 4.4 |
| 66 | 5.2 | 4 | 5.5 | 5 | 4.4 |
| 67 | 5.1 | 5 | 5.5 | 7 | 5.5 |
| 68 | 5.1 | 5 | 5.5 | 7 | 5.5 |
| 69 | 5.1 | 9 | 5.5 | 9 | 9.9 |
| 70 | 5.1 | 9 | 5.5 | 9 | 9.9 |
| 71 | 5.1 | 9 | 5.5 | 9 | 9.9 |
| 72 | 5.1 | 9 | 5.5 | 9 | 9.9 |
| 73 | 5.1 | 9 | 5.5 | 9 | 9.9 |
| 74 | 5.1 | 9 | 5.5 | 5 | 9.9 |
| 75 | 5.1 | 9 | 5.5 | 5 | 9.9 |
| 76 | 5.1 | 9 | 5.5 | 5 | 9.9 |
| 77 | 5.1 | 3 | 5.5 | 4 | 3.3 |
| 78 | 5.1 | 3 | 5.5 | 4 | 3.3 |
| 79 | 5.1 | 4 | 5.5 | 5 | 4.4 |
| 80 | 5 | 9 | 5.5 | 9 | 9.9 |
| 81 | 5 | 9 | 5.5 | 9 | 9.9 |
| 82 | 5 | 9 | 5.5 | 5 | 9.9 |
| 83 | 5 | 9 | 5.5 | 5 | 9.9 |
| 84 | 5 | 9 | 5.5 | 5 | 9.9 |
| 85 | 5 | 9 | 5.5 | 5 | 9.9 |
| 86 | 5 | 3 | 5.5 | 4 | 3.3 |
| 87 | 5 | 3 | 5.5 | 4 | 3.3 |
| 88 | 4.9 | 9 | 5.5 | 9 | 9.9 |
| 89 | 4.9 | 9 | 5.5 | 9 | 9.9 |
| 90 | 4.9 | 3 | 5.5 | 4 | 3.3 |
| 91 | 4.9 | 3 | 5.5 | 4 | 3.3 |
| 92 | 4.9 | 3 | 5.5 | 4 | 3.3 |
| 93 | 4.9 | 3 | 5.5 | 4 | 3.3 |
| 94 | 4.9 | 4 | 5.5 | 4 | 4.4 |
| 95 | 4.9 | 4 | 5.5 | 4 | 4.4 |
| 96 | 4.9 | 4 | 5.5 | 4 | 4.4 |
| 97 | 4.9 | 4 | 5.5 | 4 | 4.4 |
| 98 | 4.9 | 4 | 5.5 | 5 | 4.4 |
| 99 | 4.9 | 4 | 5.5 | 5 | 4.4 |
| 100 | 4.8 | 7 | 5.5 | 7 | 7.7 |
| 101 | 4.8 | 9 | 5.5 | 9 | 9.9 |
| 102 | 4.8 | 3 | 5.5 | 4 | 3.3 |
| 103 | 4.8 | 3 | 5.5 | 4 | 3.3 |
| 104 | 4.8 | 4 | 5.5 | 4 | 4.4 |
| 105 | 4.8 | 4 | 5.5 | 5 | 4.4 |
| 106 | 4.7 | 7 | 5.5 | 7 | 7.7 |
| 107 | 4.7 | 9 | 5.5 | 9 | 9.9 |
| 108 | 4.7 | 9 | 5.5 | 9 | 9.9 |
| 109 | 4.7 | 9 | 5.5 | 9 | 9.9 |
| 110 | 4.7 | 9 | 5.5 | 5 | 9.9 |
| 111 | 4.7 | 9 | 5.5 | 5 | 9.9 |
| 112 | 4.7 | 9 | 5.5 | 5 | 9.9 |
| 113 | 4.7 | 9 | 5.5 | 5 | 9.9 |
| 114 | 4.7 | 2 | 5.5 | 3 | 2.2 |
| 115 | 4.7 | 2 | 5.5 | 3 | 2.2 |
| 116 | 4.7 | 3 | 5.5 | 4 | 3.3 |
| 117 | 4.7 | 3 | 5.5 | 4 | 3.3 |
| 118 | 4.6 | 9 | 5.5 | 9 | 9.9 |
| 119 | 4.6 | 9 | 5.5 | 9 | 9.9 |
| 120 | 4.6 | 9 | 5.5 | 5 | 9.9 |
| 121 | 4.6 | 9 | 5.5 | 5 | 9.9 |
| 122 | 4.6 | 9 | 5.5 | 5 | 9.9 |
| 123 | 4.6 | 9 | 5.5 | 5 | 9.9 |
| 124 | 4.6 | 3 | 5.5 | 4 | 3.3 |
| 125 | 4.6 | 3 | 5.5 | 4 | 3.3 |
| 126 | 4.6 | 5 | 5.5 | 3 | 5.5 |
| 127 | 4.5 | 5 | 5.5 | 7 | 5.5 |
| 128 | 4.5 | 9 | 5.5 | 9 | 9.9 |
| 129 | 4.5 | 9 | 5.5 | 9 | 9.9 |
| 130 | 4.5 | 9 | 5.5 | 5 | 9.9 |
| 131 | 4.5 | 9 | 5.5 | 5 | 9.9 |
| 132 | 4.5 | 5 | 5.5 | 3 | 5.5 |
| 133 | 4.5 | 5 | 5.5 | 3 | 5.5 |
| 134 | 4.4 | 7 | 5.5 | 9 | 7.7 |
| 135 | 4.3 | 5 | 5.5 | 7 | 5.5 |
| 136 | 4.3 | 9 | 5.5 | 9 | 9.9 |
| 137 | 4.2 | 5 | 5.5 | 7 | 5.5 |
| 138 | 4.1 | 5 | 5.5 | 7 | 5.5 |
| 139 | 4.1 | 5 | 5.5 | 7 | 5.5 |
| 140 | 4.1 | 7 | 5.5 | 9 | 7.7 |
| 141 | 4.1 | 9 | 5.5 | 9 | 9.9 |
| 142 | 3.9 | 5 | 5.5 | 7 | 5.5 |
| 143 | 3.9 | 7 | 5.5 | 9 | 7.7 |
| 144 | 3.9 | 9 | 5.5 | 9 | 9.9 |
| 145 | 3.9 | 9 | 5.5 | 5 | 9.9 |
| 146 | 3.9 | 3 | 5.5 | 4 | 3.3 |
| 147 | 3.9 | 3 | 5.5 | 4 | 3.3 |
| 148 | 3.9 | 4 | 5.5 | 4 | 4.4 |
| 149 | 3.9 | 4 | 5.5 | 5 | 4.4 |
| 150 | 3.8 | 5 | 5.5 | 7 | 5.5 |
| 151 | 3.8 | 5 | 5.5 | 7 | 5.5 |
| 152 | 3.8 | 7 | 5.5 | 7 | 7.7 |
| 153 | 3.8 | 7 | 5.5 | 7 | 7.7 |
| 154 | 3.8 | 7 | 5.5 | 9 | 7.7 |
| 155 | 3.7 | 7 | 5.5 | 9 | 7.7 |
| 156 | 3.7 | 9 | 5.5 | 9 | 9.9 |
| 157 | 3.7 | 4 | 5.5 | 4 | 4.4 |
| 158 | 3.5 | 5 | 5.5 | 7 | 5.5 |
| 159 | 3.5 | 9 | 5.5 | 9 | 9.9 |
| 160 | 3.5 | 9 | 5.5 | 9 | 9.9 |
| 161 | 3.5 | 9 | 5.5 | 9 | 9.9 |
| 162 | 3.5 | 9 | 5.5 | 5 | 9.9 |
| 163 | 3.5 | 9 | 5.5 | 5 | 9.9 |
| 164 | 3.5 | 9 | 5.5 | 5 | 9.9 |
| 165 | 3.5 | 9 | 5.5 | 5 | 9.9 |
| 166 | 3.5 | 3 | 5.5 | 4 | 3.3 |
| 167 | 3.5 | 4 | 5.5 | 4 | 4.4 |
| 168 | 3.5 | 4 | 5.5 | 4 | 4.4 |
| 169 | 3.4 | 5 | 5.5 | 7 | 5.5 |
| 170 | 3.4 | 7 | 5.5 | 9 | 7.7 |
| 171 | 3.4 | 4 | 5.5 | 4 | 4.4 |
| 172 | 3.3 | 5 | 5.5 | 7 | 5.5 |
| 173 | 3.2 | 5 | 5.5 | 7 | 5.5 |
| 174 | 3.2 | 7 | 5.5 | 9 | 7.7 |
| 175 | 3.1 | 4 | 5.5 | 4 | 4.4 |
| 176 | 2.9 | 5 | 5.5 | 7 | 5.5 |
| 177 | 2.9 | 5 | 5.5 | 7 | 5.5 |
| 178 | 2.9 | 5 | 5.5 | 7 | 5.5 |
| 179 | 2.9 | 7 | 5.5 | 7 | 7.7 |
| 180 | 2.9 | 7 | 5.5 | 9 | 7.7 |
| 181 | 2.9 | 9 | 5.5 | 9 | 9.9 |
| 182 | 2.9 | 3 | 5.5 | 4 | 3.3 |
| 183 | 2.9 | 4 | 5.5 | 4 | 4.4 |
| 184 | 2.9 | 4 | 5.5 | 5 | 4.4 |
| 185 | 2.8 | 5 | 5.5 | 7 | 5.5 |
| 186 | 2.8 | 5 | 5.5 | 7 | 5.5 |
| 187 | 2.8 | 7 | 5.5 | 9 | 7.7 |
| 188 | 2.8 | 7 | 5.5 | 9 | 7.7 |
| 189 | 2.8 | 9 | 5.5 | 9 | 9.9 |
| 190 | 2.8 | 9 | 5.5 | 9 | 9.9 |
| 191 | 2.8 | 5 | 5.5 | 3 | 5.5 |
| 192 | 2.8 | 4 | 5.5 | 5 | 4.4 |
| 193 | 2.7 | 5 | 5.5 | 7 | 5.5 |
| 194 | 2.7 | 7 | 5.5 | 7 | 7.7 |
| 195 | 2.7 | 7 | 5.5 | 7 | 7.7 |
| 196 | 2.7 | 7 | 5.5 | 9 | 7.7 |
| 197 | 2.7 | 7 | 5.5 | 9 | 7.7 |
| 198 | 2.7 | 7 | 5.5 | 9 | 7.7 |
| 199 | 2.7 | 7 | 5.5 | 9 | 7.7 |
| 200 | 2.7 | 9 | 5.5 | 9 | 9.9 |
| 201 | 2.7 | 9 | 5.5 | 5 | 9.9 |
| 202 | 2.7 | 3 | 5.5 | 3 | 3.3 |
| 203 | 2.7 | 2 | 5.5 | 3 | 2.2 |
| 204 | 2.7 | 2 | 5.5 | 3 | 2.2 |
| 205 | 2.7 | 3 | 5.5 | 4 | 3.3 |
| 206 | 2.7 | 4 | 5.5 | 4 | 4.4 |
| 207 | 2.7 | 4 | 5.5 | 5 | 4.4 |
| 208 | 2.7 | 5 | 5.5 | 3 | 5.5 |
| 209 | 2.5 | 7 | 5.5 | 7 | 7.7 |
| 210 | 2.5 | 7 | 5.5 | 9 | 7.7 |
| 211 | 2.4 | 9 | 5.5 | 9 | 9.9 |
| 212 | 2.4 | 9 | 5.5 | 9 | 9.9 |
| 213 | 2.4 | 9 | 5.5 | 9 | 9.9 |
| 214 | 2.4 | 9 | 5.5 | 5 | 9.9 |
| 215 | 2.4 | 9 | 5.5 | 5 | 9.9 |
| 216 | 2.4 | 9 | 5.5 | 5 | 9.9 |
| 217 | 2.4 | 5 | 5.5 | 3 | 5.5 |
| 218 | 2.4 | 3 | 5.5 | 4 | 3.3 |
| 219 | 2.4 | 3 | 5.5 | 4 | 3.3 |
| 220 | 2.4 | 3 | 5.5 | 4 | 3.3 |
| 221 | 2.4 | 4 | 5.5 | 4 | 4.4 |
| 222 | 2.4 | 4 | 5.5 | 4 | 4.4 |
| 223 | 2.4 | 4 | 5.5 | 4 | 4.4 |
| 224 | 2.4 | 4 | 5.5 | 4 | 4.4 |
| 225 | 2.4 | 4 | 5.5 | 4 | 4.4 |
| 226 | 2.4 | 4 | 5.5 | 5 | 4.4 |
| 227 | 2.4 | 4 | 5.5 | 5 | 4.4 |
| 228 | 2.3 | 5 | 5.5 | 7 | 5.5 |
| 229 | 2.3 | 9 | 5.5 | 9 | 9.9 |
| 230 | 2.3 | 9 | 5.5 | 5 | 9.9 |
| 231 | 2.3 | 5 | 5.5 | 3 | 5.5 |
| 232 | 2.2 | 7 | 5.5 | 7 | 7.7 |
| 233 | 2.1 | 5 | 5.5 | 7 | 5.5 |
| 234 | 2.1 | 7 | 5.5 | 9 | 7.7 |
| 235 | 2.1 | 9 | 5.5 | 9 | 9.9 |
| 236 | 2.1 | 9 | 5.5 | 5 | 9.9 |
| 237 | 2.1 | 4 | 5.5 | 4 | 4.4 |
| 238 | 2.1 | 5 | 5.5 | 3 | 5.5 |
| 239 | 1.9 | 7 | 5.5 | 7 | 7.7 |
| 240 | 1.9 | 9 | 5.5 | 9 | 9.9 |
| 241 | 1.9 | 9 | 5.5 | 9 | 9.9 |
| 242 | 1.9 | 3 | 5.5 | 4 | 3.3 |
| 243 | 1.9 | 3 | 5.5 | 4 | 3.3 |
| 244 | 1.9 | 4 | 5.5 | 4 | 4.4 |
| 245 | 1.9 | 4 | 5.5 | 5 | 4.4 |
| 246 | 1.8 | 5 | 5.5 | 7 | 5.5 |
| 247 | 1.8 | 5 | 5.5 | 7 | 5.5 |
| 248 | 1.8 | 7 | 5.5 | 7 | 7.7 |
| 249 | 1.8 | 7 | 5.5 | 7 | 7.7 |
| 250 | 1.8 | 7 | 5.5 | 9 | 7.7 |
| 251 | 1.8 | 7 | 5.5 | 9 | 7.7 |
| 252 | 1.8 | 9 | 5.5 | 9 | 9.9 |
| 253 | 1.8 | 9 | 5.5 | 9 | 9.9 |
| 254 | 1.8 | 9 | 5.5 | 9 | 9.9 |
| 255 | 1.8 | 9 | 5.5 | 5 | 9.9 |
| 256 | 1.8 | 9 | 5.5 | 5 | 9.9 |
| 257 | 1.8 | 9 | 5.5 | 5 | 9.9 |
| 258 | 1.8 | 2 | 5.5 | 3 | 2.2 |
| 259 | 1.8 | 3 | 5.5 | 4 | 3.3 |
| 260 | 1.8 | 3 | 5.5 | 4 | 3.3 |
| 261 | 1.8 | 4 | 5.5 | 4 | 4.4 |
| 262 | 1.8 | 4 | 5.5 | 5 | 4.4 |
| 263 | 1.8 | 5 | 5.5 | 3 | 5.5 |
| 264 | 1.7 | 7 | 5.5 | 9 | 7.7 |
| 265 | 1.6 | 5 | 5.5 | 7 | 5.5 |
| 266 | 1.4 | 7 | 5.5 | 7 | 7.7 |
| 267 | 1.4 | 7 | 5.5 | 7 | 7.7 |
| 268 | 1.4 | 7 | 5.5 | 9 | 7.7 |
| 269 | 1.4 | 9 | 5.5 | 9 | 9.9 |
| 270 | 1.4 | 9 | 5.5 | 9 | 9.9 |
| 271 | 1.4 | 9 | 5.5 | 5 | 9.9 |
| 272 | 1.4 | 9 | 5.5 | 5 | 9.9 |
| 273 | 1.4 | 9 | 5.5 | 5 | 9.9 |
| 274 | 1.4 | 9 | 5.5 | 5 | 9.9 |
| 275 | 1.4 | 9 | 5.5 | 5 | 9.9 |
| 276 | 1.4 | 3 | 5.5 | 4 | 3.3 |
| 277 | 1.4 | 4 | 5.5 | 4 | 4.4 |
| 278 | 1.2 | 5 | 5.5 | 7 | 5.5 |
| 279 | 1.2 | 7 | 5.5 | 9 | 7.7 |
|  | 4.20 | 6.26 | 5.50 | 6.08 | 6.88 |

Appendix 3 – Anonymised datasets

3c - Dataset O2 weaning patients – RAG ratings & virtual ward LOS (ordered by acute LOS)

| ID | UHL LOS 1 | VW LOS | Expected UHL LOS | Number Of Green Sessions | Number Of Amber Sessions | Number Of Red Sessions | Total RAG Reports |
| --- | --- | --- | --- | --- | --- | --- | --- |
| 1 | 23.4 | 26 | 33.3 | 10 | 7 | 2 | 19 |
| 2 | 20.8 | 35 | 30.7 | 12 | 50 | 33 | 95 |
| 3 | 20.4 | 18 | 30.3 | 25 | 3 | 1 | 29 |
| 4 | 20.4 | 11 | 30.3 | 0 | 0 | 7 | 7 |
| 5 | 20.1 | 13 | 30 | 34 | 44 | 0 | 78 |
| 6 | 19.5 | 3 | 29.4 | 0 | 1 | 1 | 2 |
| 7 | 16.8 | 28 | 26.7 | 19 | 27 | 17 | 63 |
| 8 | 15.9 | 15 | 25.8 | 30 | 11 | 0 | 41 |
| 9 | 15.1 | 18 | 25 | 21 | 2 | 3 | 26 |
| 10 | 14.2 | 34 | 24.1 | 67 | 2 | 1 | 70 |
| 11 | 13.4 | 28 | 23.3 | 27 | 42 | 3 | 72 |
| 12 | 13.1 | 23 | 23 | 33 | 16 | 7 | 56 |
| 13 | 12.9 | 15 | 22.8 | 8 | 5 | 5 | 18 |
| 14 | 12.8 | 16 | 22.7 | 30 | 0 | 0 | 30 |
| 15 | 12.7 | 14 | 22.6 | 31 | 1 | 3 | 35 |
| 16 | 12.4 | 17 | 22.3 | 17 | 2 | 0 | 19 |
| 17 | 11.9 | 29 | 21.8 | 3 | 9 | 28 | 40 |
| 18 | 11.8 | 16 | 21.7 | 32 | 2 | 3 | 37 |
| 19 | 11.7 | 47 | 21.6 | 27 | 38 | 22 | 87 |
| 20 | 11.6 | 15 | 21.5 | 23 | 3 | 0 | 26 |
| 21 | 11.5 | 22 | 21.4 | 1 | 22 | 15 | 38 |
| 22 | 11.3 | 16 | 21.2 | 43 | 2 | 2 | 47 |
| 23 | 10.4 | 41 | 20.3 | 15 | 1 | 21 | 37 |
| 24 | 10.2 | 23 | 20.1 | 3 | 30 | 6 | 39 |
| 25 | 9.5 | 34 | 19.4 | 4 | 9 | 9 | 22 |
| 26 | 8.7 | 14 | 18.6 | 2 | 3 | 1 | 6 |
| 27 | 8.6 | 23 | 18.5 | 14 | 4 | 12 | 30 |
| 28 | 8.4 | 29 | 18.3 | 0 | 1 | 29 | 30 |
| 29 | 7.6 | 24 | 17.5 | 47 | 0 | 1 | 48 |
| 30 | 7.3 | 53 | 17.2 | 20 | 17 | 20 | 57 |
| 31 | 6.9 | 15 | 16.8 | 89 | 19 | 5 | 113 |
|  | 13.3 | 715 | 23.2 | 687 | 373 | 257 | 1317 |
